# 3,9-Diisopropyl-2,4,8,10-tetra­thia­spiro­[5.5]undeca­ne

**DOI:** 10.1107/S1600536810037281

**Published:** 2010-09-25

**Authors:** Şerban Andrei Gâz, Ioana Dobra, Adrian Woiczechowski-Pop, Richard A. Varga, Ion Grosu

**Affiliations:** a Organic Chemistry Department, CCSOOM, Faculty of Chemistry and Chemical Engineering, ‘Babes-Bolyai’ University, Arany Janos Street 11, RO-400028 Cluj Napoca, Romania; b Faculty of Chemistry and Chemical Engineering, ‘Babes-Bolyai’ University, Arany Janos Street 11, RO-400028 Cluj Napoca, Romania

## Abstract

The mol­ecule of the title compound, C_13_H_24_S_4_, has *C*2 symmetry and it crystallizes as a racemate. The structure displays two six-membered rings exhibiting chair conformations, with the isopropyl substituents in equatorial positions. In the crystal structure, weak inter­molecular C—H⋯S inter­actions are observed, leading to a channel-like arrangement along the *c* axis.

## Related literature

For background to the chemistry of spirans, see: Cismaş *et al.* (2005 ▶); Eliel & Wilen (1994 ▶); Grosu *et al.* (1995 ▶, 1997 ▶); Terec *et al.* (2001 ▶, 2004 ▶). For other studies regarding the synthesis and stereochemistry of spiranes bearing 1,3-dithiane units, see: Backer & Evenhuis (1937 ▶); Gâz *et al.* (2008 ▶); Mitkin *et al.* (2001 ▶). For the crystal structure of a spiran beaing 1,3-dithiane unit atoms, see: Zhou *et al.* (2001 ▶).
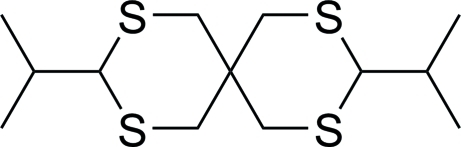


## Experimental

### 

#### Crystal data

C_13_H_24_S_4_
*M* *_r_* = 308.56
Monoclinic, 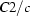

*a* = 16.701 (5) Å
*b* = 10.241 (3) Å
*c* = 12.063 (3) Å
β = 128.418 (4)°
*V* = 1616.5 (8) Å^3^
*Z* = 4
Mo *K*α radiation
μ = 0.57 mm^−1^
*T* = 297 K
0.32 × 0.31 × 0.28 mm

#### Data collection

Bruker SMART APEX CCD area-detector diffractometer
Absorption correction: multi-scan (*SADABS*; Bruker, 2000 ▶) *T*_min_ = 0.839, *T*_max_ = 0.857
7606 measured reflections
1432 independent reflections
1311 reflections with *I* > 2σ(*I*)
*R*_int_ = 0.035

#### Refinement

*R*[*F* ^2^ > 2σ(*F* ^2^)] = 0.068
*wR*(*F* ^2^) = 0.153
*S* = 1.27
1432 reflections
80 parameters
H-atom parameters constrained
Δρ_max_ = 0.36 e Å^−3^
Δρ_min_ = −0.28 e Å^−3^

### 

Data collection: *SMART* (Bruker, 2000 ▶); cell refinement: *SAINT-Plus* (Bruker, 2001 ▶); data reduction: *SAINT-Plus*; program(s) used to solve structure: *SHELXS97* (Sheldrick, 2008 ▶); program(s) used to refine structure: *SHELXL97* (Sheldrick, 2008 ▶); molecular graphics: *ORTEP-3* (Farrugia, 1997 ▶) and *DIAMOND* (Brandenburg & Putz, 2004 ▶); software used to prepare material for publication: *publCIF* (Westrip, 2010 ▶).

## Supplementary Material

Crystal structure: contains datablocks I, global. DOI: 10.1107/S1600536810037281/jh2201sup1.cif

Structure factors: contains datablocks I. DOI: 10.1107/S1600536810037281/jh2201Isup2.hkl

Additional supplementary materials:  crystallographic information; 3D view; checkCIF report

## Figures and Tables

**Table 1:** Hydrogen-bond geometry (Å, °)

*D*—H⋯*A*	*D*—H	H⋯*A*	*D*⋯*A*	*D*—H⋯*A*
C7—H7*C*⋯S1^i^	0.96	2.93	3.827 (6)	156 (1)

Symmetry code: (i) 
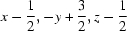
.

**Fig. 1.**
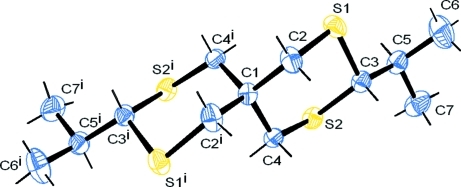
ORTEP digram of the title compound, with the atom-numbering scheme. Displacement ellipsoids are drawn at the 50% probability level and H atoms are shown as small spheres of arbitrary radii.

**Fig. 2.**
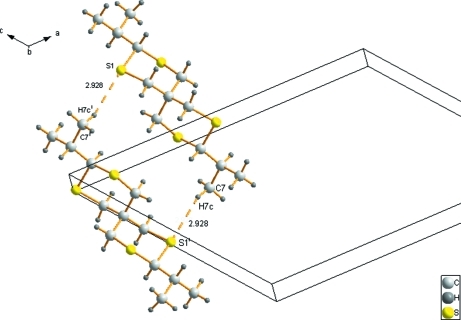
A view of the molecular structure exhibiting the hydrogen bonding interactions.

**Fig. 3.**
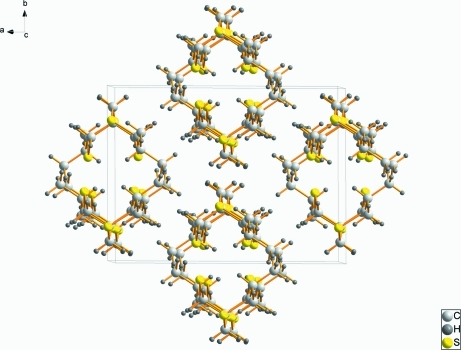
The crystal packing viewed along c axis, exhibiting channel-like arrangement formed most probably by weak interaction between the methyl group H atoms and the sulfur atom from a neighbour molecule.

## supplementary crystallographic information

### Comment

Despite the rich literature dealing with spiro compounds (Cismaş *et al.*, 2005; Eliel & Wilen, 1994; Grosu *et al.*, 1995, 1997; Terec *et al.*, 2001, 2004) new papers were written recently especially including spiro derivatives having sulfur or selenium heteroatoms. Only few spirans bearing 1,3 dithiane units were reported (Backer & Evenhuis, 1937; Gâz *et al.*, 2008; Mitkin *et al.*, 2001) and only 2 crystals were obtained so far (Zhou *et al.*, 2001). The title compound (Fig. 1) exhibits a *C*2 symmetry unit with chair conformation for both six-membered rings.

Due to the space arrangement there are differences between positions 2, 4 and 2', 4'. Due to these differencies positions 4 and 4' which are oriented towards the other 1,3-dithiane ring are named methylene inside, while the other two CH_2_ groups (positions 2 and 2') are oriented in opposite direction and they are named methylene outside groups.

In the crystal packing (Fig. 2 and Fig. 3) the sulfur atom from a neighbour molecule is hydrogen-bonded (weak interactions) *via* a intermolecular C7—H7c ···S1 connection (Table 1).

These weak interactions stabilize the lattice and form a three-dimensional network as a channel-like arrangement along the *c* axis.

### Experimental

The synthesis of I has been described elsewhere (Gâz *et al.*, 2008). Crystal were obtained from dichloromethane, by slow evaporation at room temperature.

### Refinement

All hydrogen atoms were placed in calculated positions using a riding model, with C—H = 0.93–0.97 Å and with *U*_iso_ = 1.5*U*_eq_ (C) for H. The methyl groups were allowed to rotate but not to tip.

### Crystal data

**Table:**

C_13_H_24_S_4_	*F*(000) = 664
*M**_r_* = 308.56	*D*_x_ = 1.268 Mg m^−^^3^
Monoclinic, *C*2/*c*	Melting point = 416–418 K
Hall symbol: -C 2yc	Mo *K*α radiation, λ = 0.71073 Å
*a* = 16.701 (5) Å	Cell parameters from 3441 reflections
*b* = 10.241 (3) Å	θ = 2.5–28.1°
*c* = 12.063 (3) Å	µ = 0.57 mm^−^^1^
β = 128.418 (4)°	*T* = 297 K
*V* = 1616.5 (8) Å^3^	Block, colourless
*Z* = 4	0.32 × 0.31 × 0.28 mm

### Data collection

**Table:**

Bruker SMART APEX CCD area-detector diffractometer	1432 independent reflections
Radiation source: fine-focus sealed tube	1311 reflections with *I* > 2σ(*I*)
graphite	*R*_int_ = 0.035
φ and ω scans	θ_max_ = 25.0°, θ_min_ = 2.5°
Absorption correction: multi-scan (*SADABS*; Bruker, 2000)	*h* = −19→19
*T*_min_ = 0.839, *T*_max_ = 0.857	*k* = −12→12
7606 measured reflections	*l* = −14→14

### Refinement

**Table:**

Refinement on *F*^2^	Primary atom site location: structure-invariant direct methods
Least-squares matrix: full	Secondary atom site location: difference Fourier map
*R*[*F*^2^ > 2σ(*F*^2^)] = 0.068	Hydrogen site location: inferred from neighbouring sites
*wR*(*F*^2^) = 0.153	H-atom parameters constrained
*S* = 1.27	*w* = 1/[σ^2^(*F*_o_^2^) + (0.0592*P*)^2^ + 2.605*P*] where *P* = (*F*_o_^2^ + 2*F*_c_^2^)/3
1432 reflections	(Δ/σ)_max_ < 0.001
80 parameters	Δρ_max_ = 0.36 e Å^−^^3^
0 restraints	Δρ_min_ = −0.28 e Å^−^^3^

### Special details

**Table:**

Geometry. All e.s.d.'s (except the e.s.d. in the dihedral angle between two l.s. planes) are estimated using the full covariance matrix. The cell e.s.d.'s are taken into account individually in the estimation of e.s.d.'s in distances, angles and torsion angles; correlations between e.s.d.'s in cell parameters are only used when they are defined by crystal symmetry. An approximate (isotropic) treatment of cell e.s.d.'s is used for estimating e.s.d.'s involving l.s. planes.
Refinement. Refinement of *F*^2^ against ALL reflections. The weighted *R*-factor *wR* and goodness of fit *S* are based on *F*^2^, conventional *R*-factors *R* are based on *F*, with *F* set to zero for negative *F*^2^. The threshold expression of *F*^2^ > σ(*F*^2^) is used only for calculating *R*-factors(gt) *etc*. and is not relevant to the choice of reflections for refinement. *R*-factors based on *F*^2^ are statistically about twice as large as those based on *F*, and *R*- factors based on ALL data will be even larger.

### Fractional atomic coordinates and isotropic or equivalent isotropic displacement parameters (Å^2^)

**Table:**

	*x*	*y*	*z*	*U*_iso_*/*U*_eq_	
C1	0.5000	0.7036 (5)	1.2500	0.0439 (11)	
C2	0.4969 (3)	0.6137 (4)	1.1462 (4)	0.0596 (11)	
H2A	0.4357	0.5606	1.0981	0.071*	
H2B	0.5551	0.5551	1.2001	0.071*	
C3	0.3816 (3)	0.7866 (4)	0.9229 (4)	0.0486 (9)	
H3	0.3245	0.7260	0.8842	0.058*	
C4	0.4026 (3)	0.7860 (4)	1.1697 (4)	0.0484 (9)	
H4A	0.4024	0.8353	1.2382	0.058*	
H4B	0.3444	0.7273	1.1218	0.058*	
C5	0.3655 (3)	0.8601 (4)	0.7999 (4)	0.0585 (11)	
H5	0.4243	0.9179	0.8388	0.070*	
C6	0.3597 (5)	0.7656 (6)	0.6974 (5)	0.103 (2)	
H6A	0.2997	0.7123	0.6529	0.154*	
H6B	0.4193	0.7110	0.7483	0.154*	
H6C	0.3564	0.8140	0.6266	0.154*	
C7	0.2696 (4)	0.9437 (6)	0.7207 (5)	0.0846 (16)	
H7A	0.2571	0.9797	0.6377	0.127*	
H7B	0.2786	1.0132	0.7809	0.127*	
H7C	0.2124	0.8908	0.6934	0.127*	
S1	0.49848 (9)	0.69295 (11)	1.01407 (11)	0.0627 (4)	
S2	0.38431 (7)	0.89852 (9)	1.04130 (10)	0.0529 (4)	

### Atomic displacement parameters (Å^2^)

**Table:**

	*U*^11^	*U*^22^	*U*^33^	*U*^12^	*U*^13^	*U*^23^
C1	0.050 (3)	0.036 (3)	0.041 (3)	0.000	0.026 (2)	0.000
C2	0.076 (3)	0.050 (2)	0.049 (2)	0.016 (2)	0.037 (2)	0.0054 (18)
C3	0.047 (2)	0.048 (2)	0.044 (2)	−0.0022 (16)	0.0251 (18)	0.0052 (16)
C4	0.040 (2)	0.055 (2)	0.046 (2)	−0.0010 (16)	0.0251 (17)	−0.0052 (17)
C5	0.055 (2)	0.061 (2)	0.050 (2)	−0.0053 (19)	0.028 (2)	0.0109 (19)
C6	0.139 (5)	0.112 (5)	0.061 (3)	0.014 (4)	0.064 (4)	0.018 (3)
C7	0.062 (3)	0.102 (4)	0.068 (3)	0.014 (3)	0.029 (2)	0.039 (3)
S1	0.0744 (8)	0.0695 (7)	0.0527 (6)	0.0285 (6)	0.0437 (6)	0.0142 (5)
S2	0.0497 (6)	0.0444 (6)	0.0520 (6)	0.0097 (4)	0.0254 (5)	0.0033 (4)

### Geometric parameters (Å, °)

**Table:**

C1—C4^i^	1.529 (4)	C4—H4A	0.9700
C1—C4	1.529 (4)	C4—H4B	0.9700
C1—C2	1.529 (5)	C5—C7	1.520 (6)
C1—C2^i^	1.529 (5)	C5—C6	1.524 (7)
C2—S1	1.803 (4)	C5—H5	0.9800
C2—H2A	0.9700	C6—H6A	0.9600
C2—H2B	0.9700	C6—H6B	0.9600
C3—C5	1.531 (5)	C6—H6C	0.9600
C3—S1	1.809 (4)	C7—H7A	0.9600
C3—S2	1.810 (4)	C7—H7B	0.9600
C3—H3	0.9800	C7—H7C	0.9600
C4—S2	1.798 (4)		
			
C4^i^—C1—C4	113.0 (4)	S2—C4—H4B	108.2
C4^i^—C1—C2	109.4 (2)	H4A—C4—H4B	107.4
C4—C1—C2	109.4 (2)	C7—C5—C6	109.7 (4)
C4^i^—C1—C2^i^	109.4 (2)	C7—C5—C3	111.6 (4)
C4—C1—C2^i^	109.4 (2)	C6—C5—C3	111.0 (4)
C2—C1—C2^i^	106.0 (4)	C7—C5—H5	108.2
C1—C2—S1	116.2 (3)	C6—C5—H5	108.2
C1—C2—H2A	108.2	C3—C5—H5	108.2
S1—C2—H2A	108.2	C5—C6—H6A	109.5
C1—C2—H2B	108.2	C5—C6—H6B	109.5
S1—C2—H2B	108.2	H6A—C6—H6B	109.5
H2A—C2—H2B	107.4	C5—C6—H6C	109.5
C5—C3—S1	108.9 (3)	H6A—C6—H6C	109.5
C5—C3—S2	110.9 (3)	H6B—C6—H6C	109.5
S1—C3—S2	111.59 (19)	C5—C7—H7A	109.5
C5—C3—H3	108.5	C5—C7—H7B	109.5
S1—C3—H3	108.5	H7A—C7—H7B	109.5
S2—C3—H3	108.5	C5—C7—H7C	109.5
C1—C4—S2	116.3 (2)	H7A—C7—H7C	109.5
C1—C4—H4A	108.2	H7B—C7—H7C	109.5
S2—C4—H4A	108.2	C2—S1—C3	99.99 (18)
C1—C4—H4B	108.2	C4—S2—C3	100.49 (17)
			
C4^i^—C1—C2—S1	−59.6 (4)	S1—C3—C5—C6	58.7 (4)
C4—C1—C2—S1	64.8 (4)	S2—C3—C5—C6	−178.1 (3)
C2^i^—C1—C2—S1	−177.4 (4)	C1—C2—S1—C3	−61.4 (3)
C4^i^—C1—C4—S2	58.00 (19)	C5—C3—S1—C2	−178.1 (3)
C2—C1—C4—S2	−64.2 (4)	S2—C3—S1—C2	59.1 (2)
C2^i^—C1—C4—S2	−179.8 (2)	C1—C4—S2—C3	60.4 (3)
S1—C3—C5—C7	−178.6 (3)	C5—C3—S2—C4	179.5 (3)
S2—C3—C5—C7	−55.5 (4)	S1—C3—S2—C4	−58.9 (2)

Symmetry codes: (i) −*x*+1, *y*, −*z*+5/2.

### Hydrogen-bond geometry (Å, °)

**Table:**

*D*—H···*A*	*D*—H	H···*A*	*D*···*A*	*D*—H···*A*
C7—H7C···S1^ii^	0.96	2.93	3.827 (6)	156 (1)

Symmetry codes: (ii) *x*−1/2, −*y*+3/2, *z*−1/2.
